# Medical science students’ experiences of test anxiety: a phenomenological study

**DOI:** 10.1186/s40359-022-00896-4

**Published:** 2022-07-29

**Authors:** Majid Badrian, Leila Bazrafkan, Mahsa Shakour

**Affiliations:** 1 Medical Education Department, Medical Education Development Centre, Shiraz University of Medical Sciences, Shiraz, Iran; 2 Clinical Education Research Centre, Education Developmental Centre, Shiraz University of Medical Sciences, Shiraz, Iran; 3 Educational Development Center, Arak University of Medical Sciences, Arak, Iran

**Keywords:** Test anxiety, Higher education, Coping strategies, Qualitative study

## Abstract

**Introduction:**

The studies show test anxiety is a common disorder in students that causes academic failure. There are not enough studies and specific theoretical background about test anxiety and ways to deal with it, so the purpose of this study was to do a qualitative study to fully understand the ways to deal with test anxiety in medical Sciences students.

**Materials and methods:**

This is a qualitative study. The participants are the students of the last 2 years of pharmacy, medicine and dentistry at Isfahan University of Medical Sciences. Ten students were selected by purposeful sampling, and interviews continued until the data saturation stage and the lack of access to new data. The data were analyzed by seven-level Colaizzi method.

**Findings:**

After analyzing data, about 50 codes were extracted. These codes divided into 16 subclasses, and among them, ultimately five main themes are extracted: “Prayer and Dialogue with God”, “Interaction and communication with friends and relatives”, “studying strategies”, “Finding ways to relax and self-care” and “Negative strategies” were extracted.

**Conclusions:**

The result of this study showed that students often use positive strategies to overcome the test anxiety and try to use positive strategies, but some students are advised of undesirable strategies such as misuse of authorized drugs and writing cheating that that lead to a lot of complex problems. The educational system should do its utmost effort to empower students to manage the anxiety by learning the best strategies.

## Introduction

One of the concerns of the educational system in universities is the test anxiety [1]. Test anxiety is a special type of anxiety that is characterized by physical, cognitive, and behavioral symptoms when preparing for the test and performing test, It becomes a problem when high levels of anxiety interfere with preparing for and taking the test. Test anxiety is characterized by severe fear of poor performance in tests [2]. In fact, the test anxiety is an emotional experience, feelings and anxiety in situations in which a person feels that his/her performance is evaluated [3]. Test anxiety is defined as the experience of fear, apprehension, and worry before, during, or after a test that can lead to mental distraction, memory impairment, and physical symptoms such as nausea, headache, and tachycardia [4].

This type of anxiety occurs between the ages of 10 and 12 and increases with increasing age and maximizes in higher education [5]. According to estimates by researchers in the United States, 15% of students experience different levels of anxiety annually [6]. In the study by Tsegay et al. [7] it was reported that the rate of test anxiety in medical students was 52.3% and significantly higher in female students. Test anxiety is important because it affects test success [8]. Test anxiety can cause academic failure by reducing intrinsic motivation and existing cognitive ability [9]. Sarason [10] considers test anxiety as a self-preoccupation, which is characterized by self-defeat and doubt about his/her abilities. this anxiety often leads to negative cognitive assessment, undesirable physiological reactions and the decline of academic performance. As a result, there is a significant reverse relationship between anxiety scores and test scores [10]. Various studies have shown that test anxiety has a significant and important effect on the academic performance of students. Cassady [11] showed in his study that students with higher test anxiety had a weaker academic performance [11].

Students try to cope with anxiety in order to manage it. Coping is the effort to control and manage situations that seem dangerous and tense [12]. There are two main strategies for coping with anxiety that are known as problem-focused coping and emotion-focused coping strategies. The problem-focused coping strategy in which the main objective is to dominate the position and change in the source of anxiety, and emotion-focused coping strategies in which the main objective is to reduce or transform the emotional disturbance quickly [13].

Narimany and et al. stated that students manage their anxiety through conventional methods such as: they think about good memories of the past, praying and eliminating negative thoughts and beliefs [14].

In previous studies, descriptive studies have focused more on estimating the extent to which positive methods have been used, and less on the overall evaluation of all coping strategies that students use. Also, in this study, an attempt has been made to study the strategies of medical students to deal with test anxiety in Eastern and Islamic countries. In Eastern countries, beliefs are different from some other countries where previous studies have been conducted, so its findings can help improve knowledge in this area. On the other hand, this study has selected medical students as the target population because these students face a high volume of difficult. They may also choose different strategies to deal with test anxiety. In this regard, the present study was designed in qualitative study in this target group and conducted with the aim of identifying strategies to deal with test anxiety in medical students.

## Materials and methods

This study is qualitative. The qualitative research method adopted for this study is interpretative phenomenological analysis (IPA), because IPA is a qualitative thematic approach developed within psychology, focusing on the subjective lived experiences of individuals. The participants were students of pharmacy, medicine and dentistry at Isfahan University of Medical Sciences. A purposeful method was used for targeted sampling. Inclusion criteria included studying in the last 2 years of dentistry, medicine and pharmacy, as well as having the desire to participate in the study. Sampling was continued until data saturation.

This study received ethical approval from the Institutional Review Board (IRB) to which the researchers are affiliated. All study protocols were performed in accordance with the Declaration of Helsinki. This study considered ethical considerations such as the confidentiality of the interviewees’ names and the written consent of interviewees. Interviews were conducted in 2020. Informed consent of every participant was obtained after clearly explaining the objectives as well as the significance of the study for each participant. We advised the participants about the right to participate as well as refuse or discontinue participation at any time they want and the chance to ask anything about the study. The participants were also advised that all data collected would remain confidential.

Data collection was done in a semi-structured interview. The questions were about the test anxiety manage strategies used by students. Interviews continued until data saturation and lack of access to new data. Interview questions were semi-structured and probing questions were asked. Every interview lasted roughly between 30 and 70 min. Interviews were recorded by audio tape and at the earliest opportunity verbatim transcription of interview data was done. Statements were written word-by-word and then were manually coded.

Data was analyzed by the seven-level Colaizzi method. The Colaizzi steps were performed as follows; (1) Transcribing all the participants’ descriptions. participant narratives transcribed from the audio-taped interviews. We didn’t use any software. (2) Extracting significant statements, statements that directly relate to the test anxiety. The researchers repeated all participants’ descriptions and in order to understand these concepts, it was felt by them, then extracted the sentences and vocabulary related to the phenomenon under study and gave a special meaning to each of the extracted sentences. (3) Creating formulated meanings. In this stage, each significant statement is extracted from the participant’s narratives. (4) Aggregating formulated meanings into theme clusters. We organized formulated meanings into groups of similar type. (5) Developing an exhaustive description. An exhaustive description developed through a synthesis of all theme clusters and associated formulated meanings explicated by the researchers. (6) After articulation of the symbolic representation which occurred during the interview. Researchers did an interpretative analysis of symbolic representations for test anxiety. (7) We tried to identify the fundamental structure of the test anxiety by explication’ through a rigorous analysis of the exhaustive description of it.

In order to ensure the accuracy of the data, rigor and trustworthiness was determined based on Guba and Lincholn criteria (1994) which include Credibility, Dependability, Confirmability and Transferability [15, 16]. Therefore, we used member checking, researcher creditability, prolonged engagement (semi-structured interview) with the participants, the use of peer debriefing. A follow-up appointment was made between the researcher and each participant for the purpose of validating the essence of the phenomenon with students.

In order to get rigor and trustworthiness data, we established comfortable interactions at the beginning of the interviews, which was maintained until the end of the interview. Participants were also surveyed about the codes for approval after each interview. Data, coding and themes were also reviewed by an expert in this subject.

## Findings

Ten students participated in the study, of which 7 persons were females and 3 persons were males. Students were selected of pharmacy, medicine and dentistry.

After analyzing the data, about 50 codes were extracted. These codes are divided into 16 subclasses, and among them, ultimately, five main themes, called “prayer and dialogue with God”, “Interaction and communication with friends and relatives”, “studying strategies”, “Finding ways to relax and self-care” and “Negative strategies” were extracted.

*Prayer and dialogue with God:* Pray to God and trust in God led to the extraction of this theme. Participant No. 3 stated: “I am asking the mother to pray for me to take my test successfully and reduce my anxiety”. Participant No. 7 stated that “I believe in the Jafar e Tayyar prayers (it is a special continuous prayer for one’s requests from God) and at night before of test, and I would be very calm”.

Participant No. 1 stated that “I already have a good relationship with the Quran (Islam’s book), and I read a Quran page before the test and it calmed me”.

The other subtheme was trusting in God. A student on this subtheme stated that “I vow about tests that I have a lot of anxiety, and this creates peace in me”.

Participant No.3 said: “I trust in God and I ask him to help me, and thereby keep calm down and begin studying with greater focus”.

*Interaction and communication with friends and relatives*: This theme was included two subthemes: “communication with the family” and “communication with friends”. Participant No. 4 about communication with the family said, “I talk to my parents over the phone at the test night in the dormitory, and they will calm me with their words”.

On communication with friends, Participant No. 10 stated: “I talk to friends who are very intimate and express my anxiety and this reduces my anxiety”. Participant No. 2 said, “I talk to classmates who have a joint test, and talk about the test. This will reduce my anxiety”.

*Studying strategies*: This theme includes two subthemes: “More effort in education” and “Applying different study strategies”. For “Studying earlier” Participant No. 1 stated that “I’ve been studying for weeks before the test, this will reduce my anxiety”.

Participant No. 4 about using the different strategies of study, said that “I will try to study with other friends (group study) for any test that I have anxiety”. Participant No. 3 stated: “I am studying similar questions to reduce my anxiety”. Participant No. 7 said that; “Reading the summary of the important content that other friends extracting from my booklet and book reduces my anxiety”.

*Finding ways to relax and self-care*: it was another major theme that was included subthemes: “relaxing activities”, “exercise”, and “consuming Caffeinated beverages”.

Regarding this theme, Participant No. 5 stated that; “When I have anxiety due to the test, I try to listen to my favorite music. This will manage my thoughts and also do not sleep”. Or Participant No. 1 said that; “Walking in the open air helps me become more fluent and calm down”. Participant No. 3 noted that “I usually drink coffee or Nescafe when I have hard and difficult test, this will reduce my anxiety”.

“*Negative strategies*” This theme has two subthemes: “drug abuse” and “rely on cheating”. Participant No. 9 about rely to the cheating said that “I am cheating to reduce the anxiety of the test, even if I do not intend to use it, this makes calm down me”. Participant No. 8 about drug abuse stated that, “I’m eating a Propranolol 10 when I having an overwhelming anxiety”. Participant No. 6 stated that, “I used the Ritalin tablet when I fear from test and anxietyed me, although it did not work as a result of my test”.

## Discussion

In the present study, with a qualitative approach and interviews with students, researchers tried to identify and describe the experiences of strategies for coping with the anxiety of the test among students in dentistry, medicine and pharmacy.

One of the main themes that students use to cope test anxiety is “prayer and dialogue with God”. Students use prayer and dialogue with God to reduce their anxiety. Among the religious and spiritual sources, the greatest source used to reduce anxiety and anxiety is “prayer”. The prayer is derived from the Latin word of precarious meaning “obtained by pray and pleasure” [17]. The other studies showed praying is an effective strategy to cope with test anxiety. Adroishi et al. [18] showed that listening to pray could significantly reduce the test anxiety in students. The study of Masomi et al. showed that listening to the Quran sound, such as the sound of music before the test, is an effective strategy for the anxiety test, and the Quran sound is more effective in reducing student test anxiety [19]. In a study conducted by Narimany et al., 44.5% and 27.5% of the students used the use of prayer as a strategy for coping with anxiety [14]. Also, training religious values [20] and spiritual training, including prayer, forgiveness, transcendental and spiritual meditation [21] have also been reported to reduce the depression and anxiety of students. Ganji et al. also reported that religious beliefs are related with anxiety levels and reduce it [22]. In Papazisis et al. research, strong religious and spiritual beliefs have positive relationships increasing, the self-confidence, and a negative relationship with depression, anxiety and anxiety as a personality trait [23].

Based on the findings of various studies, spiritual health determines the integrity of the person, and is the only force that harmonizes the physical, psychological and social dimensions. Religious and spiritual beliefs make a person calm and play a vital role in adapting to tension [24]. Most people believe that the influence of uncontrollable positions can be controlled through relay to God [25]. Religion, spirituality, and existential health are important predictors of mental health [26, 27].

Another strategy for students to calm down and manage their anxiety was to communicate with those who had some kind of attachment to them. In fact, this communication with people such as parents or intimate friends has made them relaxed and helped them better control the tension of the test. Cassidy and Shaver, in their justification of the relationship between attachment style and mental health, stated that the consequence of a safe attachment process is creating a sense of safety in a person, and the consequence of an unsafe attachment process is to create fear in a person [28]. Roberts et al. in their justification of this relationship, believe that the psychological consequence of unsafe attachment styles in tension situations is anxiety and depression. The psychological consequence of a safe attachment style in such situations is mental relaxation [29]. By studying 314 surviving adults from Bam earthquake, Rahimian Bouger et al. found that there was a significant positive relationship between safe attachment style and mental health, and a significant negative relationship between avoidance and ambivalent attachment styles with mental health [30]. The study of Besharat et al. revealed that the subjects with a safe attachment style rather than an unsafe type and those with an avoidant attachment style had fewer interpersonal problems than ambivalent styles. The results of this study refer to authenticity of safe attachment to the first requirement and its transfer to subsequent generations [31]. Safford has shown that people with an unsafe attachment style are more likely to experience anxiety and depression [32].

Morey and Taylor [33] study, which was similar to the present study in terms of quality with in-depth interviews, found that exercise and talking to friends are two important strategies for students to deal with stress. Fujii [34] reported that one of the ways to deal with English test anxiety in students is “cooperation with others” and “building confidence”.

*Studying strategies* which were students’ strategies to cope with test anxiety, can be considered as a kind of sensitization to manage anxiety and manage of the situation. These strategies are effective and positive strategies that can help the student in a constructive way to gain more control and to manage negative thoughts, and reduce his own anxiety through such actions. Motevalli et al. [35] reported that teaching new and practical study skills helps students manage test anxiety. They found that learning some skills such as time managing for studying, properly review and summarize, how to answer multiple-choice questions, correct/incorrect and descriptive questions can control test anxiety [35]. Yusefzadeh et al. [36] reported that based on the findings of a training and evaluation program (teacher 10–35% of the final grade of the course is based on activity during the semester), the implementation of such programs significantly reduces anxiety Students are tested. Ozbiçakçi et al. [37] also believe that teaching method reduces test anxiety. But L. Hsu mentioned that further research is needed to determine best practices for alleviating student stress and anxiety [38].

“*Finding ways to relax and self-care*” is the other main theme for coping with test anxiety. It included Relaxing activities, consuming caffeine and Soothing food. Findings of the study by Mojarrab et al. [39] showed that the use of relaxation techniques along with playing soothing music reduces nursing students’ test anxiety and also improves their clinical test scores. Morey and Taylor [33] reported that many students believed exercise would distract them from test anxiety and lead to be calm. Walking, running and yoga were among the activities that students focused on, with more emphasis on walking [33].

In this study, *negative strategies* were also used to manage the anxiety by the students, such as *Unusual use of some medications*. Evidences show that the consumption of stimulants and non-drug use of a variety of drugs is one of the threats to students. Several evidences suggest that consuming drug abuse among young prople, especially students, is increasing. Methylphenidate or Ritalin is one of the most widely used drugs that has recently been abused among adolescents, especially students [40]. Many students take this medicine to stay awake for several hours and to maintain their unusual focuses for a long time. During the tests, the use of such drugs, including Ritalin, increases. Ritalin is the most common prescribed psychotropic drug for children in the USA [14]. Currently, non-medical consumption of prescription stimulants, including Methylphenidate, is a growing problem among students in the USA. Statistics have shown that 7% of American students have at least once used this drug over their lifetime, and the prevalence of Methylphenidate consumption among students over the course of 1 year was 3% [13]. In this regard, several studies have reported 3–35% of Ritalin abuse among students [41–46]. The most common side effects of consuming Ritalin are insomnia, nervousness, anxiety, headache, and loss of appetite. In excessive consumption, restlessness, delirium, psychosis, hypertension, seizure, hyperthermia, and arrhythmias may occur [47].

*Writing cheating* was one of the other negative strategies that students used to reduce their test anxiety, although they had no intention of using this cheating during the test. The student, by writing cheat, wants to create a reliance point on himself/herself and thereby calm himself down and overcome his anxiety. In fact, cheating as a reliance point of its existence is an important part of assurance, not to applying it.

## Conclusions

The result of this study showed that students often use positive strategies to overcome the test anxiety and try to use positive strategies, but some students are advised of undesirable strategies such as misuse of authorized drug and writing cheating that lead to a lot of complex problems. Given that the test are an important part of academic life and that every student is always involved in a test and study evaluation, the educational system should do its utmost effort to empower students to manage anxiety by learning the best strategies.

## Data Availability

The datasets used and analyzed during the current study are not publically available due to ethical restriction and personal data protections but are available from the corresponding author on reasonable request.
